# Acute effects of caffeine and cigarette smoking on ventricular long-axis function in healthy subjects

**DOI:** 10.1186/1476-7120-6-9

**Published:** 2008-03-04

**Authors:** Elisa Giacomin, Elisabetta Palmerini, Piercarlo Ballo, Valerio Zacà, Giovanni Bova, Sergio Mondillo

**Affiliations:** 1 Department of Cardiovascular Disease, University of Siena, Italy; 2 Cardiology Operative Unit, S. Andrea Hospital, La Spezia, Italy

## Abstract

**Background:**

Few data exist regarding the direct effects of caffeine and smoking on cardiac function. We sought to explore the acute effects of caffeine assumption, cigarette smoking, or both on left ventricular (LV) and right ventricular (RV) function in a population of young normal subjects.

**Methods:**

Forty-five healthy subjects aged 25 ± 2 years underwent echocardiography. Fifteen of them were non-smokers and habitual coffee consumers (group 1), 15 were smokers and not habitual coffee consumers (group 2), and 15 were smokers and habitual coffee consumers (group 3). Peak systolic (S_a_), early diastolic E_a_, and late diastolic (A_a_) velocity of mitral annulus were measured by pulsed Tissue Doppler, and left atrioventricular plane displacement was determined by M-mode. Tricuspid annular velocities and systolic excursion (TAPSE) were also determined. Measurements were performed at baseline and after oral assumption of caffeine 100 mg in group 1, one cigarette smoking in group 2, and both in group 3.

**Results:**

No changes in ventricular function were observed in group 1 after caffeine administration. In group 2, cigarette smoking yielded an acute increase in mitral A_a _(+12.1%, p = 0.0026), tricuspid S_a _(+9.8%, p = 0.012) and TAPSE (+7.9%, p = 0.017), and a decrease in the mitral E_a_/A_a _ratio (-8.5%, p = 0.0084). Sequential caffeine assumption and cigarette smoking in group 3 was associated with an acute increase in mitral A_a _(+13.0%, p = 0.015) and tricuspid A_a _(+11.6%, p < 0.0001) and a reduction in mitral E_a_/A_a _ratio (-8.5%, p = 0.0084) tricuspid E_a _(-6.6%, p = 0.048) and tricuspid E_a_/A_a _ratio (-9.6%, p = 0.0003). In a two-way ANOVA model controlling for hemodynamic confounding factors, changes in the overall population remained significant for mitral A_a _and E_a_/A_a _ratio, and for tricuspid A_a _and E_a_/A_a _ratio.

**Conclusion:**

In young healthy subjects, one cigarette smoking is associated to an acute impairment in LV diastolic function and a hyperdynamic RV systolic response. Caffeine assumption alone does not exert any acute effect on ventricular long-axis function, but potentiates the negative effect of cigarette smoking by abolishing RV supernormal response and leading to a simultaneous impairment in both LV and RV diastolic function.

## Background

Both caffeine assumption and cigarette smoking are well known to yield considerable changes in cardiovascular hemodynamics. Increases in blood pressure and heart rate related to dietary intake of caffeine and cigarette smoking [1-3] as well as reductions after short-time abstinence [4,5] have been previously reported. Caffeine assumption has also been shown to acutely increase both systolic and diastolic blood pressure [6] with no significant changes in heart rate [7,8], whereas an adrenergic-mediated acute increase in blood pressure and heart rate has been observed after cigarette smoking [9]. Moreover, available data support the hypothesis that caffeine assumption and cigarette smoking may show synergistic effects on the hemodynamic status [10,11].

Nonetheless, few evidences exist regarding the direct effects of caffeine and smoking on cardiac function. Studies on animal models suggest that intravenous caffeine may acutely affect left ventricular (LV) relaxation without altering contractility [12], although a depression in invasively determined indices of systolic and diastolic LV function has been observed after intracoronary administration [13]. Caffeine was found to have no effects on standard indices of diastolic and short-axis systolic function after oral administration in humans [14]. Both acute and chronic smoking have been reported to induce LV diastolic impairment in normal subjects [15,16], an effect that seems to be more evident in type-2 diabetes patients [17]. Abnormalities in right ventricular (RV) diastolic performance after acute or chronic exposition to cigarette smoke have also been reported [18,19]. However, whether caffeine administration and cigarette smoking may have a synergistic impact on LV cardiac function has never been assessed.

The aim of this study was to analyze the acute effects of caffeine assumption, cigarette smoking, or both, on LV and RV performance in a population of young healthy subjects.

## Methods

### Study population

The study population included a total of 45 young healthy volunteers (mean age 25 ± 2 years) free of cardiovascular or systemic diseases. Of them, 15 were non-smokers and habitual coffee consumers (group 1), 15 were smokers and not habitual coffee consumers (group 2), and 15 were smokers and habitual coffee consumers (group 3). Ten non-smokers and non habitual coffee consumers were considered as controls. None of patients were assuming any medication.

### Study protocol

#### Baseline measurements

Informed written consent for the participation to the study was obtained from all participants. All subjects were asked to abstain from smoking, coffee, and other foods or beverages containing caffeine (e.g., tea, cola, cacao, guarana) for a wash-out period of at least 12 hours before examinations. Baseline systolic blood pressure, diastolic blood pressure, and heart rate were measured after 5 minutes of resting in the supine position, using standard procedures. Arterial oxygen saturation was also measured using a digital pulse oximeter.

Echocardiographic examinations were performed using high-quality machines (Vivid 7, GE, USA) equipped with 2.5 MHz probes. LV diameters and thicknesses, LV mass, end-diastolic LV relative wall thickness, end-diastolic RV diameter were determined in accordance with current ASE recommendations [20]. LV volumes, stroke volume, and ejection fraction were measured using the biplane modified Simpson's method. Left atrial volume was obtained from apical views using the biplane method of discs. Pulsed Doppler interrogation of mitral inflow was performed to measure peak early diastolic velocity (E), peak late diastolic velocity (A), their ratio E/A, E wave deceleration time, and isovolumic relaxation time. Mitral annulus velocities were measured using pulsed Tissue Doppler by positioning a 5 mm-sample volume at the level of septal, lateral, inferior and anterior annulus, in accordance with current ASE recommendations [21]. Particular care was given to adjust filter and gain settings at the minimal level to obtain the maximal signal-to-noise ratio. The average value of peak systolic (S_a_), early diastolic (E_a_), and late diastolic (A_a_) mitral annulus velocities were determined. The E_a_/A_a _and E/E_a _ratios were calculated, and used as indices of LV filling pressures [22-24]. Pulsed Tissue Doppler imaging of the lateral tricuspid annulus was also performed, and peak systolic, early diastolic, and late diastolic velocities were measured. Two-dimensionally guided M-mode imaging of septal, lateral, inferior, and anterior mitral annulus motion was performed from the apical 4-chamber view, using the zoom function. Total amplitude of systolic annular excursion was measured from the nadir of M-mode profile – corresponding to the point furthest from LV apex – to the point of maximal excursion towards LV apex [25]. Left atrioventricular plane displacement (AVPD) was determined by averaging excursion amplitudes recorded at the four annular sites. Tricuspid annular plane systolic excursion (TAPSE) was also measured using two-dimensionally guided M-mode imaging from the apical 4-chamber view. For both Tissue Doppler and M-mode imaging, careful alignment of the ultrasonic beam with annular motion was obtained. All measurements were obtained by averaging values recorded in three consecutive cycles, at a sweep speed of 100 mm/s.

#### Caffeine assumption and cigarette smoking

**Figure 1:**
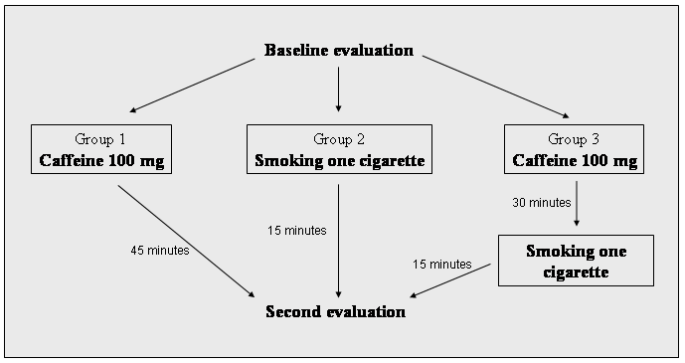
Study design.

The study design is summarized in Figure 1. At the end of baseline evaluation, subjects in group 1 were asked to assume caffeine 100 mg administered orally (galenic preparation solved in 40 ml of water), a dosage that is equivalent to that of an express coffee. Subjects in group 2 were asked to smoke one cigarette in 5 minutes. Subjects in group 3 were asked to assume caffeine 100 mg per os, to wait for 30 minutes, and then to smoke one cigarette in 5 minutes. Cigarettes used for groups 2 and 3 contained nicotine 0.9 mg, carbon monoxide 10 mg, and tar 10 mg, and were of the same brand (Camel).

A second clinical and echocardiographic examination was performed after a total of 45 minutes from caffeine assumption in group 1, after 15 minutes from beginning of cigarette smoking in group 2, and after 45 minutes from caffeine assumption (i.e., 15 minutes from beginning of cigarette smoking) in group 3. During these periods, patients were asked to rest quiet in the sitting position. The second examination included measurement of systolic blood pressure, diastolic blood pressure, heart rate, arterial oxygen saturation, mitral inflow indices, mitral annulus velocities and AVPD averaged over four annular sites, lateral tricuspid annulus velocities, and TAPSE.

**Figure 2:**
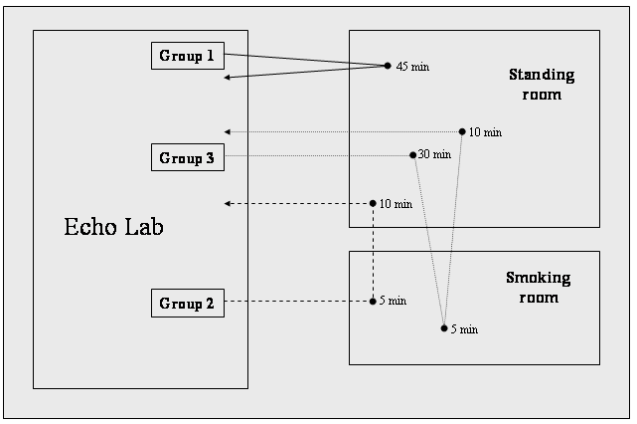
Experimental setting of the study. Caffeine in group 1 and 3 was administered within the Echo Lab, at the end of basal examination.

The experimental setting (Figure 2) included a smoking room and a standing room, both characterized by quiet and comfortable environments. The smoking room was only used by patients in group 2 and 3 for 5 minutes during cigarette smoking. The standing room was used for all other protocol intervals.

### Statistical analysis

Data were expressed as mean ± standard deviation. Between-group comparisons at baseline were performed using the Kruskal-Wallis test for continuous variables and the chi-square test for categorical variables. Within-group comparisons between baseline values and those obtained after caffeine assumption, cigarette smoking, or both were performed using the Student t test for paired samples. Repeated measures two-way ANOVA was also performed by considering groups and status (i.e., baseline or after testing) as the main variables within a 3 × 2 factorial design, using a mixed model adjusting for heart rate, systolic blood pressure, and diastolic blood pressure. The P value for the main effect of status was used to express the significance of the effect of caffeine and smoking on ventricular function in the overall population, whereas the interaction P value was considered to explore for differences in the effect of testing across groups. A P value < 0.05 was considered for statistical significance. All tests were two-tailed. Analyses were performed using the SPSS (Statistical Package for the Social Sciences, Chicago, Illinois) software Release 11.5.

## Results

Baseline clinical and echocardiographic characteristics were similar among the four study groups (Table 1). Changes in clinical variables and mitral flow Doppler indices after caffeine assumption, cigarette smoking, or both in comparison with baseline are shown in Table 2. An increase in systolic blood pressure, diastolic blood pressure, and heart rate was observed in group 2. An isolated increase in heart rate was found in group 3, whereas no changes were noted in group 1.

**Table 1:** General characteristics of the study groups. Comparison of clinical and echocardiographic features among the four study groups.

	**Controls**	**Coffee assumption**	**Cigarette smoking**	**Coffee assumption + cigarette smoking**	**ANOVA P value**
**Age **(years)	24.1 ± 1.7	25.3 ± 4.3	24.9 ± 2.7	23.6 ± 2.5	0.68
**Male gender **(n)	6 (60%)	9 (60%)	6 (40%)	9 (60%)	0.68
**BMI **(Kg/m^2^)	20.2 ± 2.1	20.7 ± 5.5	21.0 ± 1.7	22.0 ± 2.4	0.38
**Coffees **(n/die)	-	2.5 ± 1.1	-	3.0 ± 2.4	0.28*
**Cigarettes **(n/die)	-	-	8.4 ± 5.3	11.7 ± 5.1	0.09*
**Years of smoking **(n)	-	-	7.5 ± 3.8	8.1 ± 2.7	0.66*
**SBP **(mmHg)	116.0 ± 8.1	125 ± 9.6	116.7 ± 12.8	122.3 ± 2.5	0.11
**DBP **(mmHg)	75.0 ± 5.8	76.7 ± 9.6	75.0 ± 11.2	79.7 ± 7.9	0.50
**Heart rate **(bpm)	74.9 ± 10.0	74.5 ± 10.6	72.7 ± 11.4	66.1 ± 9.4	0.15
**O_2 _saturation **(%)	97.5 ± 1.2	97.6 ± 0.7	97.9 ± 0.7	97.5 ± 0.9	0.77
**LVEDV **(ml)	89.0 ± 28.2	99.7 ± 28.3	89.4 ± 14.2	92.9 ± 29.5	0.84
**Ejection fraction **(%)	67.4 ± 3.2	64.8 ± 3.6	64.2 ± 5.5	63.8 ± 4.8	0.14
**Stroke volume **(ml)	60.0 ± 18.5	66.4 ± 23.0	57.8 ± 5.5	59.2 ± 17.4	0.89
**LV mass **(g)	126.6 ± 31.4	132.0 ± 45.1	122.1 ± 22.5	143.2 ± 40.0	0.40
**RWT**	0.32 ± 0.05	0.32 ± 0.05	0.34 ± 0.05	0.34 ± 0.07	0.82
**Left atrial volume **(ml)	42.6 ± 11.4	38.8 ± 16.2	34.0 ± 7.5	38.6 ± 10.4	0.34
**RV diameter **(mm)	30.7 ± 4.2	31.3 ± 5.4	29.3 ± 4.3	31.12 ± 4.3	0.60
**PASP **(mmHg)	20.5 ± 2.7	21.9 ± 3.6	21.6 ± 3.4	21.2 ± 4.5	0.66
**E **(cm/s)	80.2 ± 19.3	92.1 ± 14	89.1 ± 14.8	84.1 ± 13.0	0.32
**A **(cm/s)	52.2 ± 15.5	54.2 ± 11	53.9 ± 8.9	48.2 ± 13.2	0.42
**E/A ratio**	1.6 ± 0.3	1.8 ± 0.4	1.7 ± 0.4	1.9 ± 0.5	0.47
**Deceleration time **(ms)	209.8 ± 32.7	201.9 ± 50.3	202.5 ± 35.9	216.9 ± 34.8	0.70
**IVRT **(ms)	62.2 ± 9.4	67.6 ± 12.8	67.1 ± 10.7	63.1 ± 10.4	0.49

BMI = body mass index. SBP = systolic blood pressure; DBP = diastolic blood pressure; LVEDD = left ventricular end-diastolic diameter; LV = left ventricular; RWT = relative wall thickness; RV = right ventricular; PASP = pulmonary artery systolic pressure; IVRT = isovolumic relaxation time.

*P values for comparison of variables between two groups calculated by Student t test for unpaired data

**Table 2:** Clinical variables and mitral inflow after coffee assumption, cigarette smoking, or both. Comparison of clinical and echocardiographic characteristics among groups after coffee assumption, cigarette smoking, or both. Abbreviations are the same used in Tables 1–2.

	**Coffee assumption**		**Cigarette smoking**		**Coffee assumption and cigarette smoking**		**Adjusted overall P value^b^**	**Interaction P value^c^**
	
	**Change from baseline**	**P value^a^**	**Change from baseline**	**P value^a^**	**Change from baseline**	**P value^a^**		
**SBP **(mmHg)	+1.2%	0.43	+8.6%	0.0010	+4.8%	0.17	-	-
**DBP **(mmHg)	+3.2%	0.24	+7.6%	0.013	+3.3%	0.30	-	-
**HR **(bpm)	-3.2%	0.19	+18.7%	< 0.0001	+14.5%	< 0.0001	-	-
**SO_2 _**(%)	-0.2%	0.63	0.3%	0.36	+0.2%	0.58	0.62	0.52
**E **(cm/s)	-6.1%	0.11	+2.6%	0.80	+0.7%	0.99	0.26	0.15
**A **(cm/s)	-8.3%	0.06	-2.0%	0.79	+9.3%	0.81	0.29	0.22
**E/A ratio**	+4.8%	0.19	+10.8%	0.49	-2.7%	0.71	0.68	0.39
**DT **(ms)	+5.1%	0.99	-1.9%	0.76	-7.8%	0.15	0.34	0.28
**IVRT **(ms)	+3.8%	0.60	+7.1%	0.41	+5.1%	0.36	0.28	0.92

^a ^Calculated by within-group comparison with baseline value

^b ^P value for the overall effect of testing in the entire population, as determined by two-way ANOVA using a mixed model adjusting for heart rate and blood pressure

^c ^P value for differences in the effect of testing across groups, as determined by two-way ANOVA in a mixed model adjusting for heart rate and blood pressure

**Figure 3:**
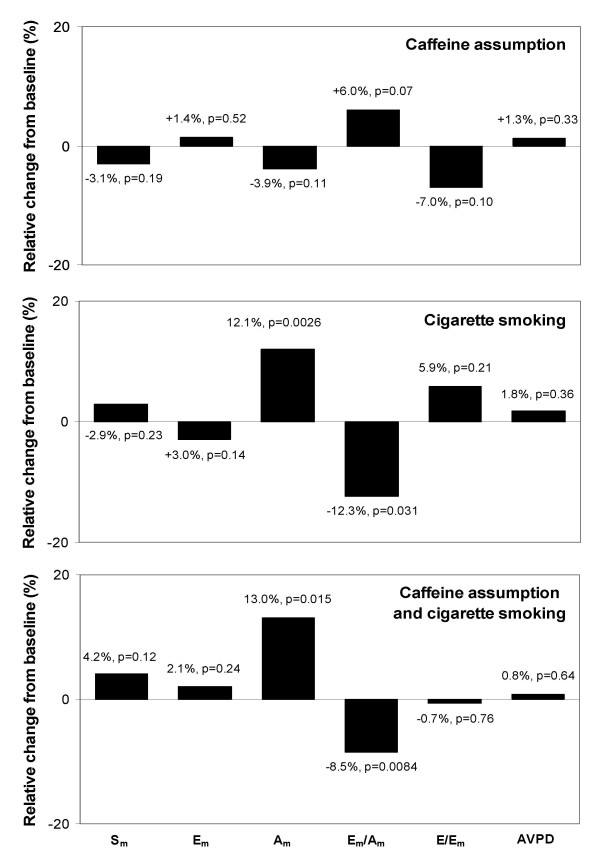
Relative changes in left ventricular long-axis function after caffeine assumption, cigarette smoking, or both. P values are calculated by within-group Student t test for paired data. S_m _= peak systolic mitral annulus velocity; E_m _= peak early diastolic mitral annulus velocity; A_m _= peak late diastolic mitral annulus velocity; E = peak early diastolic transmitral flow; AVPD = left atrioventricular plane displacement.

**Figure 4:**
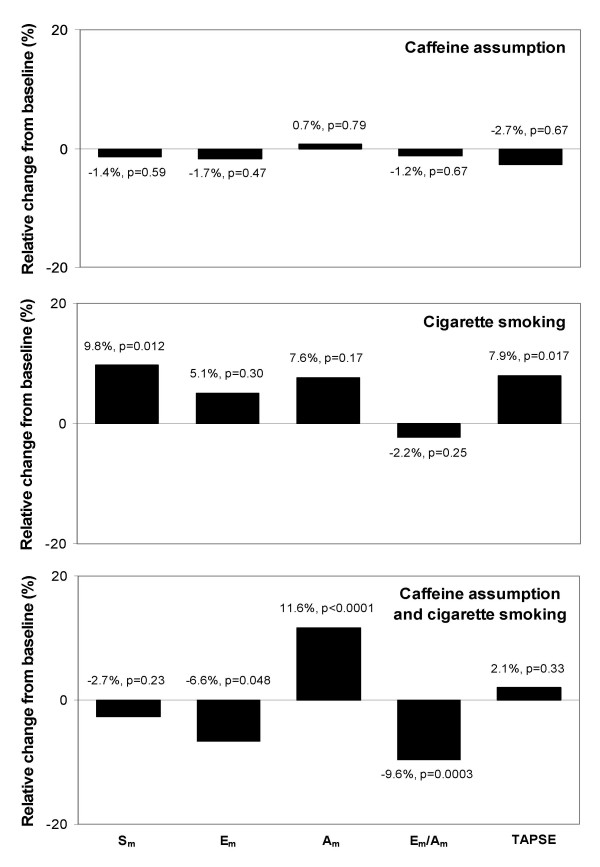
Relative changes in right ventricular long-axis function after caffeine assumption, cigarette smoking, or both. P values are calculated by within-group Student t test for paired data. S_m _= peak systolic tricuspid annulus velocity; E_m _= peak early diastolic tricuspid annulus velocity; A_m _= peak late diastolic tricuspid annulus velocity; TAPSE = tricuspid annular plane systolic excursion.

At within-group analysis, caffeine assumption in group 1 did not yield any change in any of the analyzed variables (Figures 3, 4, top panels). In group 2, an increase in mitral A_a_, tricuspid S_a_, and TAPSE, and a reduction in the mitral annulus E_a_/A_a _ratio were observed after cigarette smoking (Figures 3, 4, middle panels). In group 3, an increase in mitral A_a _and tricuspid A_a_, and a reduction in mitral E_a_/A_a _ratio, tricuspid E_a_, and tricuspid E_a_/A_a _ratio were observed after sequential caffeine assumption and cigarette smoking (Figures 3, 4, bottom panels).

In a two-way ANOVA model adjusting for hemodynamic confounding factors, changes in long-axis function observed in the overall population remained significant for mitral A_a _(p = 0.0014) and E_a_/A_a _ratio (p = 0.0022), and for tricuspid A_a _(p = 0.010) and E_a_/A_a _ratio (p = 0.019). For these 4 variables, significant interaction P values were found (p = 0.0009, p = 0.0003, p < 0.0001, and p = 0.0006, respectively), suggesting differences in the effect of testing across groups. Significant interaction P values were also observed for tricuspid S_a _(p = 0.038) and TAPSE (p = 0.049).

## Discussion

The results of this study show that: 1) caffeine assumption does not yield any significant change in ventricular long-axis function; 2) cigarette smoking is associated with an acute impairment in LV diastolic function and an increase in RV long-axis systolic indices; 3) caffeine assumption followed by cigarette smoking is associated with an acute impairment in both LV and RV diastolic function.

**Figure 5:**
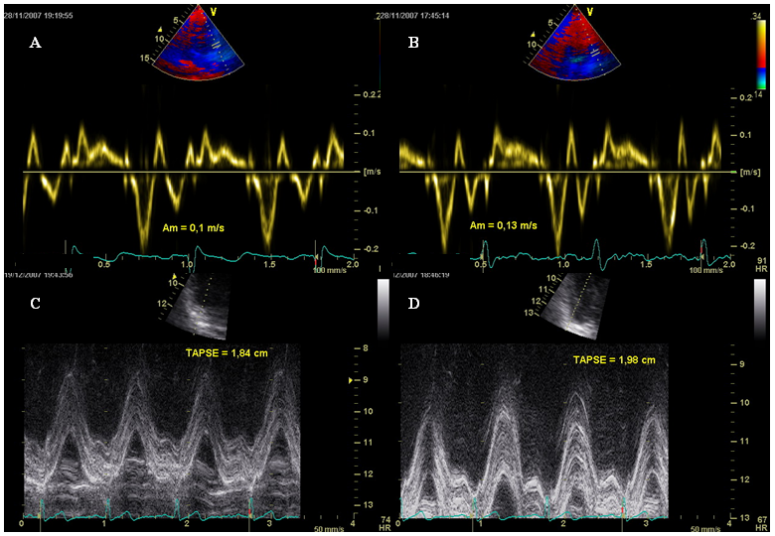
Tissue Doppler pattern of mitral annulus motion and M-mode imaging of tricuspid annulus motion at baseline (panels A-C) and after cigarette smoking (panels B-D), showing an increase in mitral peak A_m _velocity and tricuspid annular plane systolic displacement (TAPSE).

**Figure 6:**
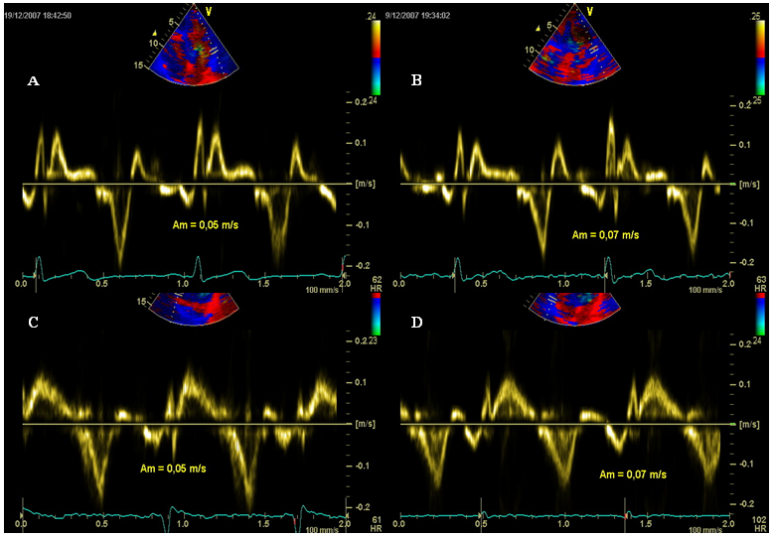
Tissue Doppler pattern of mitral and tricuspid annulus motion at baseline (panels A-C) and after coffeine assumption followed by cigarette smoking (panels B-D), showing an increase in both mitral and tricuspid peak A_m _velocities.

The absence of significant effects of caffeine administration on LV long-axis indices observed in this study confirms early reports that found no effects on standard indices of LV systolic and diastolic function [26], and strengthens this hypothesis by showing that functional changes after caffeine assumption cannot be detected even when sensitive indices of ventricular function are considered. The evidence of changes in diastolic long-axis indices after cigarette smoking is also in accordance with current available data [16,27]. Of note, in our population most of this effect was driven by an increase in the late diastolic component of mitral annulus motion (Figure 5, top panels), with only a nonsignificant trend towards a reduction in the early diastolic component. This resulted in a 12% reduction in the mitral E_a_/A_a _ratio – an index of LV diastolic function that correlates with LV filling pressures [23]. Moreover, in this study caffeine assumption followed by cigarette smoking was associated with an increase in mitral A_a _and a reduction in the E_a_/A_a _ratio similar to that observed after cigarette smoking alone (Figure 6, top panels). This may indicate that caffeine assumption does not alter the acute effect of cigarette smoking on LV function, suggesting that the synergistic effect between smoke and caffeine previously demonstrated for blood pressure [11,28,29] cannot be extended to LV function.

Considering that nicotine gum chewing does not yield any acute change in LV diastolic performance in young healthy individuals [30], it is likely that the acute effect of smoking on LV diastolic function may not be due to nicotine alone. A potential role of smoke-induced acute impairment in coronary blood flow could be hypothesized [26,31]. Additionally, the absence of significant changes in the E/E_a _ratio – a powerful index of LV filling pressure [24] – suggests the effective mechanisms underlying the observed changes in diastolic mitral annulus velocities deserve further investigations.

In this study, caffeine assumption showed no acute effect on RV function as well. In contrast, cigarette smoking was associated with an acute increase in indices of RV long-axis systolic function (Figure 5, bottom panels). It could be hypothesized that this effect may represent the consequence of an acute adrenergic stimulus induced by nicotine [32,33]. Nonetheless, an interaction between neuroendocrine factors and variations in RV afterload due to acute changes in the vascular tone at the level of pulmonary vascular bed [19] cannot be excluded. Intriguingly, the hyperdynamic response of RV long-axis systolic function was no longer evident in patients assuming caffeine prior to cigarette smoking. Moreover, in these subjects an impairment of RV diastolic indices, characterized by an increase in the late diastolic component of tricuspid annulus motion and a reduction in the tricuspid E_m_/A_m _ratio, was observed (Figure 6, bottom panels). The mechanisms underlying these changes are worthy of further exploration. However, these results may suggest that caffeine and cigarette smoking could exert a synergistic negative effect on RV function.

The findings of this study further highlight the importance of smoking as a cardiovascular risk factor, by pointing out that its well-known deleterious effects on the cardiovascular system include a depression in LV diastolic performance that is already evident few minutes after smoking a single cigarette. Moreover, the evidence that the association of caffeine administration and cigarette smoking resulted in simultaneous impairment in both LV and RV diastolic function suggests that concomitant assumption of coffee (or other food or beverages containing caffeine) and cigarette smoking should be avoided.

This study has limitations. A lager sample size would have added statistical solidity to our results. The study population included young healthy subjects, so that caution is required in extending results to other populations. The choice of an interval of 45 minutes between baseline and second examination in group 1 and 3 was based on a general estimate of the time necessary to reach the peak plasmatic concentration of caffeine after oral assumption, but a considerable inter-individual variability in caffeine kinetics exists [34]. Similar considerations can be made for the arbitrary choice of an interval of 15 minutes after the beginning of smoking. Assessment of LV and RV diastolic function was performed using Tissue Doppler indices, but further studies on invasively determined measures of ventricular function are needed.

## Conclusion

In summary, one cigarette smoking acutely impairs LV diastolic function and is associated to a hyperdynamic RV systolic response in young normal subjects. Concomitant caffeine assumption may enhance the negative effect of cigarette smoking by abolishing RV hyperdynamic response and favouring simultaneous impairment in both LV and RV diastolic performance.

## Competing interests

The author(s) declare that they have no competing interests.

## Authors' contributions

All authors participated to the design of the study. EP, EG, GB, and VZ were responsible for collection of data and drafting of manuscript. PB performed the statistical analysis and revised the manuscript critically for important intellectual content. SM conceived the study and revised the manuscript critically for important intellectual content. All authors read and approved the final manuscript.
